# Legal, Moral and Political Determinants within the Social Determinants of Health: Approaching Transdisciplinary Challenges through Intradisciplinary Reflection

**DOI:** 10.1093/phe/phaa009

**Published:** 2020-05-03

**Authors:** John Coggon

**Affiliations:** Centre for Health, Law, and Society and Bristol Population Health Science Institute, University of Bristol

## Abstract

This article provides a critical analysis of ‘the legal’ in the legal determinants of health, with reference to the *Lancet*–O’Neill report on that topic. The analysis shows how law is framed as a fluid and porous concept, with legal measures and instruments being conceived as sociopolitical phenomena. I argue that the way that laws are grounded practically as part of a broader concept of politics and evaluated normatively for their instrumental value has important implications for the study of law itself. This, in turn, has implications for how we approach the transdisciplinary ambitions that form a key part of the report’s recommendations to enhance law’s capacity to promote better, more equitable population health at local, national, international and global levels.

## The Legal Determinants of Health and the *Lancet*–O’Neill Report: Background and Framing

This article critically explores ‘the legal’ in the legal determinants of health, as represented in the *Lancet*–O’Neill Institute of Georgetown University Commission report (the *Lancet*–O’Neill report) entitled *The Legal Determinants of Health: Harnessing the Power of Law for Global Health and Sustainable Development* (Gostin *et al.*, 2019). The report, whose lead author is the pioneering public and global health lawyer Lawrence Gostin, is a commendable contribution to efforts better to shape structural factors that impact the public’s health at local, national, international and global levels. It is a critical work that looks to the power of law: law’s strong determinative effects, for better and for worse, on our social, governmental, commercial and physical environments; and their consequent impacts on health and health inequalities. And the report is an aspirational and directive work: it looks to law’s place and potential in public and global health agendas to achieve fairer and healthier societies. In the authors’ words:

This *Lancet* Commission articulates the crucial role of law in achieving global health with justice, through legal instruments, legal capacities, and institutional reforms, as well as a firm commitment to the rule of law. The Commission’s aim is to enhance the global health community’s understanding of law, regulation, and the rule of law as effective tools to advance population health and equity. (Gostin *et al.*, 2019: 1857)

The *Lancet*–O’Neill report advances recommendations that together aim to extend and refine law’s *capacity* to protect health, promote well-being and reduce health inequalities within and across nations. The Commission argues that this capacity may be realized through three functions that law might lend to the methods of social coordination and collective activity that are central to assuring the conditions for good and equitable health in ways that cannot be realized through individual, or individual-focused, efforts (*cf*Verweij and Dawson, 2009). These arise from: first, law’s prospective function in establishing standards and norms to guide different actors and agencies; second, its methods of dispute resolution that clarify and enforce obligations, as well as serving policy agendas through strategic litigation efforts; and third, its functions in strengthening the governance of public and private institutions (Gostin *et al.*, 2019: 1863–1868).

One component of the capacity building that the report recommends concerns the links between the study of law and its consequent incorporation in public and global health agendas, research and practice. In particular, the report emphasizes the problems caused by disciplinary silos and by the relative rarity of expertise that spans law and public/global health:

Researchers and practitioners in law and in health have traditionally worked in quite distinct ways. In the medical profession, as in the wider public, understanding or recognition of the power of law to drive behavioural and social change is lacking. […] For their part, lawyers can be protective of their turf and unwilling to acknowledge the limits of their subject-matter expertise. This silo mentality leads to missed opportunities for teaching, research, practice, and problem-solving. (Gostin *et al.*, 2019: 1898)

Given such concerns about monodisciplinary isolationism, my aim here is to contribute reflections from the perspective of a legal scholar with interests in the philosophy, practice and politics of public and global health. I do so with reference to the different characterizations of law (and thus legal determinants) within the *Lancet*–O’Neill report and with reference to the rationalizations of its recommendations concerning practical and academic engagement with law. The repeated characterization of law as a *tool*—as *per* the opening quotation in this article—presents law as something to be understood within an agenda that spans sectors and systems, rather than as a concept or practice that may usefully—meaningfully, even—be isolated or understood in purely abstract terms. This leads to the view that ‘the legal’ in the legal determinants of health is best conceived as a fluid and porous idea. It requires to be understood in context and over time. Insofar as this permits an *intra*disciplinary legal perspective, it is one that would shape the study of law as something that itself embraces varied and contingent methods and points of reference. Such an understanding of law suggests that lawyers’ ‘subject-matter expertise’ must extend, on its own terms, beyond technical and procedural questions concerning laws themselves. Whilst it would be complacent to allow this to nullify concerns about ‘protecting turf’ or obstacles to working across professions and disciplines, a broader and less cohesive appreciation of law as a single discipline lends itself well to the transdisciplinary ambitions of the *Lancet*–O’Neill report.

To examine the points of synergy and disconnect between law(s), justice and healthy outcomes, it is useful to explore and explain how law itself is conceptualized by the *Lancet*–O’Neill Commission. In the remainder of this article, I will argue that as the characterization of law moves from a ‘purer’ concept to something more porous and varied, so its disciplinary boundaries weaken and its internal coherence, or disciplinary purity, diminishes. I accordingly refer to the *idea* of law: following (after a fashion) Amartya Sen’s approach to understanding justice through plural grounding and non-ideal theory (*cf*Sen, 2009), I consider it both normatively and practically preferable not to work with a singular, pure account of law, but rather to allow for legal determinants to cover a range of radically distinct ideas and forms. I will show that whilst laws are explained in the *Lancet*–O’Neill report as having distinguishing features both formally and institutionally, they are ultimately conceived as sociopolitical phenomena: they are *part of* a broader array of mechanisms that represent sociopolitical power and its exercise.

## The Idea of Law in the *Lancet*–O’Neill Report

On its face, the *Lancet*–O’Neill report works with a contained concept of law that, whilst socially contingent, may be represented as appealing to features of a pure or prototypical understanding of what law and legal systems are, and how they impact society. The Commission writes:

The term law throughout is used to mean legal instruments such as statutes, treaties, and regulations that express public policy, as well as the public institutions (eg, courts, legislatures, and agencies) responsible for creating, implementing, and interpreting the law. By establishing the rules and frameworks that shape social and economic interactions, laws exert a powerful force on all the social determinants of health. (Gostin *et al.*, 2019: 1857)

In this initial framing, law is presented as a distinct and particularly potent source of power, control and coordination. And the distinguishing features of laws, and of legal actors and agencies, are ones that we might associate with concepts found in socially grounded theories of legal positivism; notably, those focused on the domestic legal systems of sovereign states (*cf*Hart, 1994). Domestic legal systems are commonly perceived as exhibiting stronger practical and normative coherence than is found in international law and legal systems. This is so in particular given national systems’ (generally) clearer constitutional bases, the more practically and conceptually coherent nature of their institutional arrangements and the consequently greater rigour of their claims to authority and effectiveness. International legal measures and instruments might be said to be ‘less like law’ than domestic ones: they exhibit, so this position holds, fewer of the features and institutional arrangements with reference to which we identify something as law.

Despite, therefore, its opening representation of law, and thus what would constitute legal determinants, as the report progresses, we come to find the embrace of broader, less pure ideas of law. This is both reasonable and unsurprising given the *Lancet*–O’Neill Commission’s focus on global health. As noted, accommodation of measures and instruments within international law that do not enjoy all of the defining trappings of domestic laws accords with many jurisprudential traditions and assumptions (Fidler, 2008; Coggon, 2014). It conforms too with the distinct emphases and framing in Gostin’s definition of global health law in his independent work, which is more firmly rooted in concepts of *governance* (Gostin, 2014), as contrasted with Gostin and Lindsay Wiley’s definition of (national) public health law, which more rigidly focuses on prototypical concepts of law and legal form (Gostin and Wiley, 2016). It bears stressing, having made these points, that the *Lancet*–O’Neill report’s overall emphasis on the functions of law (outlined in the opening section of this paper) and its instrumentalist approach to understanding and evaluating law mean that there is an embrace of governance within the legal determinants of health, both at domestic and international/transnational/global levels (see also Coggon *et al.*, 2017: chapter 4).

## ‘The Legal’ within the Political Determinants of Health

The approach taken in the *Lancet*–O’Neill report, which does not limit its concerns to structural and regulatory features that are born of strict legal form, means that the conceptual reach of legal determinants both pervades and is pervaded by matters that are not strictly law, but which by analysis require to be understood if law itself is to be understood. I will argue that this means that legal determinants are integrated within the political determinants of health: that is the overall aspects of power, control and coordination that reside (or arguably should reside) with governmental actors and agencies (legal and otherwise) (see also Coggon, 2012: chapter 11).

The placing of legal determinants within political determinants is most starkly represented through the *Lancet*–O’Neill Commission’s self-linking to the report of the earlier *Lancet*–University of Oslo Commission’s report on Global Governance for Health (the *Lancet*–Oslo report) (Ottersen *et al.*, 2014). The *Lancet*–O’Neill report says that it ‘builds on’ (Gostin *et al.*, 2019: 1859) the work of that earlier Commission. In so doing, it explains the overlaps between law and governance, whilst emphasizing that governance has a wider embrace: ‘Law is central to governance, but governance goes beyond law’ (Gostin *et al.*, 2019: 1877). We may accept this categorization, whilst also accepting that the plural understandings of law within the *Lancet*–O’Neill report mean that law itself is a diffuse and ranging idea rather than in an entirely separable analytical (and practical) class with its own firm borders. Governance is a broad and multifarious categorization; law is a graded and pluralistic one that sits within it.

The overall reach of the ‘more diffuse concept’ (Gostin *et al.*, 2019: 1877) of governance is explained by the *Lancet*–O’Neill Commission’s quoting Thomas Weiss and Ramesh Thakur’s definition, which was adopted by the *Lancet*–Oslo Commission. They characterize global governance as:

The complex of formal and informal institutions, mechanisms, relationships, and processes between and among states, markets, citizens, and organisations, both intergovernmental and non-governmental, through which collective interests on the global plane are articulated, rights and obligations are established, and differences are mediated. (Thakur and Weiss, 2006)

The *Lancet*–Oslo report directly addresses the *political* determinants of health. It ‘examines power disparities and dynamics across a range of policy areas that affect health and that require improved global governance’ (Ottersen *et al.*, 2014: 630). The *Lancet*–O’Neill Commission emphasizes how law may provide greater rigour to efforts in governance for health (note especially the third function of law listed in the opening section of this article). However, the legal in the legal determinants of health cannot be approached only with understanding of ‘pure’ instances of law. This is because, first, legal governance is *part of* governance writ large. Second, the idea of legal itself enjoys an expansive and pluralistic understanding. And third, law (writ narrow)’s strengths, weaknesses and ultimate capacity cannot be understood without an understanding of the wider social and governance contexts. Legal determinants may be less diffuse than all of the political determinants, but they are still diffuse and of the same kind. Such framing of legal determinants is reinforced if we consider both how the *Lancet*–O’Neill Commission invites law to be understood as a grounded, sociopolitical phenomenon, and how its normative evaluations of and prescriptions for law are determined by extra-legal, moral considerations.

Regarding the first point, the *Lancet*–O’Neill report urges that laws be conceived as lived, evolving, empirical phenomena: they cannot be understood in the abstract or in isolation, and their meaning is found and changes in context and over time. The Commission writes:

Enacting a good health law is only the first step towards building an effective legal environment. Laws that are defined as on the books must be supported by effective processes for their drafting (including public participation), implementation, enforcement, monitoring, evaluation, and ultimately their revision or repeal where necessary[.] (Gostin *et al.*, 2019: 1894)

If a scholar’s or practitioner’s understanding of law were limited to contained (siloed) expertise, she could not on this count understand law. Expertise in law requires capacities that extend beyond technical legal knowledge; a point that supports calls for *transdisciplinary* approaches to public and global health law. In a study cited in the *Lancet*–O’Neill report, Burris *et al.* (2016: 138) demonstrate how public health law does not exist exclusively ‘within the professional jurisdiction of lawyers’. In explaining their ideas, they focus in particular on *legal epidemiology*, which examines the population health effects of law, legal practice and policy (so again not limited to a pure account of law) by combining the practical and epistemological resources and traditions of law with those of epidemiological research.

Such transdisciplinarity is crucial, but in developing the capacity for this, there are important lessons too for legal scholarship and studies in an *intra*disciplinary sense. The indications in the report of what law *is*, and what is required to study and practise law, undermine any meaningful case that might be made for narrow or siloed approaches to legal studies. Insofar as this is not already recognized and reflected in practice (and there will be variation within and across different academic traditions; *cf*Bartie, 2010; Siems and Mac Síthigh, 2012), law as a discipline itself requires to be (re)configured in a way that is not (in principle) consistent with its being a silo. At the levels of analysis and practice, the report promotes the idea of law as an area of expertise that contains distinct approaches and methods. These naturally fall within the traditions of critical, socio-legal studies (for discussion of socio-legal approaches in the context of health and law, see Farrell *et al.*, 2017). Reflecting on law in this way supports the *Lancet*–O’Neill Commission’s transdisciplinary ambitions within public and global health law:

Building the empirical evidence base for effective health laws first requires building disciplinary bridges: mutual understanding, collaboration, common terminologies, and an appreciation of how different skill sets can be applied to public health problems. It also requires genuine interdisciplinary (or even transdisciplinary) research, drawing on the expertise of legal scholars, epidemiologists, clinical scientists, policy analysts, behaviour change experts, and anthropologists, amongst others, working together. (Gostin *et al.*, 2019: 1901)

However, wider philosophical as well as empirical understanding is needed. Moving to law’s normative foundations and our critical evaluations of it, I would urge that the above ideas be taken further, both as regards intradisciplinary reflection on law, and in terms of the disciplinary reach that public and global health agendas aim to achieve. Burris *et al.* (2016: 139) give some attention to ‘normative framing and analysis’, and the *Lancet*–O’Neill report has justice and commitments to democratic ideals of human rights and the rule of law written through to its core. However, a much more prominent role for (political) philosophy and ethics requires to be built into agendas at all levels for the public’s health. This becomes clear when we see how the foundational and directive idea of ‘good laws’ within the *Lancet*–O’Neill report is not to be understood by narrow reference to legal form or procedure, or the technical soundness of legal argument. Nor is it to be understood by reference (say) to natural law positions that rest on necessary conceptual links between ‘central cases of law’ and theories of morality (*cf* e.g. Finnis, 1980).

In contradistinction with jurisprudential studies that focus on bare, formalist concerns of what law is and how it works, or a core concept of law defined by its necessary relationship with morality, the *Lancet*–O’Neill Commission drives its conceptual understandings of law in the ways explained above, whilst evaluating and establishing the proper use of law by reference to moral ideas found in health and justice. The report operates on the basis that law may be variously understood, but it is consistently approached with regard to its utility; its strengths as a tool. The moral evaluations themselves might be seen as built on, and indeed constrained by, fundamental democratic ideals embodied by human rights and the rule of law; foundational concepts in legal philosophy. But they draw from health-focused considerations in justice too. These might be characterised as deriving, for example, from claims that health is a fundamental moral value (see e.g. Gostin, 2008; Gostin and Wiley, 2016: chapter 1). And they might—additionally or alternatively—be seen as being informed by accounts of the moral foundations of public health practice, which tend to be rooted in combined concerns for protecting and improving health and reducing unfair health inequalities (see Coggon and Viens, 2017). In relation to each of these, the moral determinants *in* health lead to claims about governments’ responsibilities *for* the public’s health; i.e. they represent (possible) moral foundations to the political (including legal) determinants of health and require to be analyzed and evaluated within such a framing (see Coggon, 2012: 277–283). Crucially, both in terms of legal studies and public and global health agendas, the normative foundations are not understood by exclusive reference to the internal workings or epistemologies of a ‘pure’ understanding of law.

In keeping with the *Lancet*–O’Neill Commission’s rationales and recommendations, my push for the significance of philosophical contributions is one that invites their inclusion in concert with approaches and insights from other disciplines (see further Coggon, 2012: chapter 7). This includes disciplines within the health sciences, but also other sources of critical theory, and areas such as anthropology, political sciences and sociology. Moral and broader philosophical argumentation alone will not be *sufficient* to motivate sociopolitical (including legal) changes to the determinants of (ill) health (Gostin, 2008; Coggon, 2014), but it is *necessary* to the rigour of the claims that would underpin imperatives to improve health (howsoever understood) and advance particular concepts of equity. And this necessity does not end with abstract claims about justice: as indicated in other papers in this journal issue, critical attention is required in identifying the lens through which we scrutinize questions of equity in relation to public and global health, and in looking at the implications and consequences (intended and unintended) of different social situations, policies and proposals for intervention and reform (Gangoli, 2020; Hawkes and Buse, 2020; McGuinness and Montgomery, 2020). The aspirations towards global health *with justice* require ethical justification that can both challenge harmful aspects of the *status quo* and support arguments for what should replace it, and within what normative constraints. A scientific evidence base may be crucial to the rigour of public health law, but the work of legal determinants must have broader foundations than (say) epidemiology (Horton, 2018; Venkatapuram and Bibby, 2018): it extends as well to include bases in approaches to identifying and responding to concerns through the methods of political philosophy; in debates on justice. And—again in keeping with the *Lancet*–O’Neill report’s recommendations—such forms of analysis will need to continue over time and be subject to revision in their detail as circumstances and understandings change.

## Conclusions: Laws as Means; Better, Fairer Global Health as an End

Seeing the legal determinants of health as part of the political determinants, seeing the drivers for change as coming from separable moral concerns and working with a fluid and porous concept of ‘the legal’ mean that pure instances of law arise to be measured alongside other methods and systems of governance. Nothing in these observations serves to deny that law may bring moral as well as practical authority and underpinnings to questions of social ethics, including those concerning the public’s health: that is why the *Lancet*–O’Neill report (rightly) identifies the vital importance of promoting fundamental ideals such as the rule of law, equality before the law, human rights and the empowering effect of the right to health. Nevertheless, the framing in the report provides that the sovereignty of nation states is the source of their authority, including their law-making authority. It is from that authority, the Commission argues, that states’ duty to protect and promote the public’s health arises; a duty that is underscored by the inability of individuals acting alone to do so (Gostin *et al.*, 2019: 1862–1863). Accordingly, the report can be seen as separating normative ideas such as health as a moral value, social justice and the rule of law on the one hand, from more practically grounded points where laws and their effects are seen as empirical aspects of sociopolitical realities on the other hand. In these latter regards, empirical observations and analysis of power dynamics and influence provide real-world context and themselves shape laws; and our understandings of law.

The broad range of approaches to comprehending and analysing ‘the legal’ in the legal determinants of health is well captured in the following summary:

The most just and effective public health laws share the following four core characteristics: they are evidence based, equity promoting, multisectoral, and supported by good governance[.] (Gostin *et al.*, 2019: 1882)

Seeing laws in broad terms, and evaluating them by reference to their effects rather than (say) their form or institutional (or even constitutional) foundations, lends itself naturally to the inter- and transdisciplinary points that the *Lancet*–O’Neill Commission recommends should pervade educational agendas in public and global health. If we accept the view that ‘law is only a tool, and its effectiveness depends on how this tool is used’ (Gostin *et al.*, 2019: 1889), we move to a position where legal studies, and the legal determinants of health, neither start nor end with ‘the law’. As well as providing reasons to knock down barriers to working between disciplines, this has significant implications for the discipline of law itself.
